# Effects of aerobic exercise training in oxidative metabolism and mitochondrial biogenesis markers on prefrontal cortex in obese mice

**DOI:** 10.1186/s13102-022-00607-x

**Published:** 2022-12-16

**Authors:** Matheus Santos de Sousa Fernandes, Felipe J. Aidar, Anderson Apolônio da Silva Pedroza, Severina Cássia de Andrade Silva, Gabriela Carvalho Jurema Santos, Rafael dos Santos Henrique, Filipe Manuel Clemente, Ana Filipa Silva, Raphael Fabrício de Souza, Diorginis José Ferreira, Georgian Badicu, Claudia Lagranha, Hadi Nobari

**Affiliations:** 1 Graduate Program in Neuropsychiatry and Behavioral Sciences, Center for Medical Sciences, Federal University of Pernambuco, Recife, Pernambuco Brazil; 2 Department of Physical Education, Federal University of Sergipe, São Cristovão, Sergipe Brazil; 3 Laboratory of Biochemistry and Exercise Biochemistry, Department of Physical Education and Sports Science, Federal University of Pernambuco, Vitória de Santo Antão, PE Brazil; 4 Department of Nutrition, Federal University of Pernambuco, Recife, Brazil; 5 Department of Physical Education, Federal University of Pernambuco, Recife, Brazil; 6 Escola Superior Desporto e Lazer, Instituto Politécnico de Viana do Castelo, Rua Escola Industrial e Comercial de Nun’Álvares, 4900-347 Viana do Castelo, Portugal; 7 Instituto de Telecomunicações, Delegação da Covilhã, 1049-001 Lisbon, Portugal; 8 Research Center in Sports Performance, Recreation, Innovation and Technology (SPRINT), 4960-320 Melgaço, Portugal; 9 Department of Physical Education, Federal University of São Francisco Valley, Petrolina, Pernambuco Brazil; 10 Department of Physical Education and Special Motricity, Transilvania University of Brasov, 500068 Brasov, Romania; 11 Department of Motor Performance, Faculty of Physical Education and Mountain Sports, Transilvania University of Braşov, 500068 Brasov, Romania; 12 Department of Exercise Physiology, Faculty of Educational Sciences and Psychology, University of Mohaghegh Ardabili, Ardabil, 56199-11367 Iran; 13 Faculty of Sport Sciences, University of Extremadura, 10003 Cáceres, Spain

**Keywords:** Obesity, Physical exercise, Aerobic exercise, Oxidative stress, Mitochondria, Brain

## Abstract

**Background:**

To evaluate the effects of 8 weeks of Aerobic Physical Training (AET) on the mitochondrial biogenesis and oxidative balance in the Prefrontal Cortex (PFC) of leptin deficiency-induced obese mice (ob/ob mice).

**Methods:**

Then, the mice were submitted to an 8-week protocol of aerobic physical training (AET) at moderate intensity (60% of the maximum running speed). In the oxidative stress, we analyzed Malonaldehyde (MDA) and Carbonyls, the enzymatic activity of Superoxide Dismutase (SOD), Catalase (CAT) and Glutathione S Transferase (GST), non-enzymatic antioxidant system: reduced glutathione (GSH), and Total thiols. Additionally, we evaluated the gene expression of PGC-1α SIRT-1, and ATP5A related to mitochondrial biogenesis and function.

**Results:**

In our study, we did not observe a significant difference in MDA (*p* = 0.2855), Carbonyl’s (*p* = 0.2246), SOD (*p* = 0.1595), and CAT (*p* = 0.6882) activity. However, the activity of GST (*p* = 0.04), the levels of GSH (*p* = 0.001), and Thiols (*p* = 0.02) were increased after 8 weeks of AET. Additionally, there were high levels of PGC-1α (*p* = 0.01), SIRT-1 (*p* = 0.009), and ATP5A (*p* = 0.01) gene expression after AET in comparison with the sedentary group.

**Conclusions:**

AET for eight weeks can improve antioxidant defense and increase the expression of PGC-1α, SIRT-1, and ATP5A in PFC of ob/ob mice.

## Background

Obesity is a chronic metabolic disease that affects more than a billion people worldwide [1]. Characterized by individuals with body mass index (BMI) higher than 30 kg/m^2^, obesity has been listed as a risk factor for several chronic degenerative diseases [2]. Studies have demonstrated a variety of factors associated with the establishment of obesity, such as genetic, hormonal, and behavioral [3–5], wherein it has been described their relationship with areas of the Central Nervous System (CNS) that regulate energy balance and food intake [6, 7].

Moreover, it has been demonstrated that obesity might disturb cognitive centers by impairing neural networks in the Prefrontal Cortex (PFC) [8]. The PFC is responsible for high executive functions such as attention, motor planning, and working memory, as well as the regulation of the limbic reward [9]. Due to the interaction between the limbic system and emotional regulation, PFC significantly influences food intake's behavioral control. Previous studies in the literature suggest that unhealthy eating behaviors result in poor executive functioning of the PFC, resulting in compromised self-control [10]. Since dorsolateral PFC plays a role in the supervision of eating and making healthy eating decisions, additional studies show that obese individuals have less left dorsolateral PFC activation after a meal compared with lean individuals, indicating dysfunction in the inhibitory mechanisms responsible for the control of the eating behavior and food choice [11]. In this context, obesity affects executive functions assessed through Delay Discounting, Penn Progressive Matrices, Picture Vocabulary, and Dimensional Change Card Sort Tests [12–15].

Contrariwise, Aerobic Physical Training (AET) [16–18] has been widely used in the prevention and treatment of obesity as well as in the enhancement of brain function, like neuromuscular rehabilitation protocol [19]. As a metabolic enhancer, exercise can improve lipolysis, immunity, oxidative metabolism, neuroplasticity, as well as mitochondria function, and oxidative stress resilience [20].

Oxidative stress is the process that can be defined as an imbalance between the production of the Reactive Oxygen Species (ROS) and their elimination [21, 22]. According to data in literature, several neurodegenerative diseases (e.g., Alzheimer, Parkinson, Huntington, and Multiple sclerosis) are associated with higher levels of ROS and the establishment of oxidative stress [21, 23, 24]. In the same way, recent data in the literature suggest the important role of ROS and oxidative stress in the brain dysfunctions associated with obesity [25, 26].

Despite the data in the literature demonstrating that exercise can reduce the deleterious effects induced by obesity on the brain, interestingly, up to now, there is a gap in the literature evaluating the specific effect of moderate aerobic exercise in a specific area of the brain, responsible for taking of decisions, and control of executive’s patterns linking oxidative stress and mitochondrial biogenesis. Therefore, the present study aims to evaluate in PFC from ob/ob mice the levels of oxidative stress and the gene expression of mitochondrial biogenesis (i.e., PGC1α, SIRT-1, and ATP5A).

## Methods

### Ob Ob mice model

Male mice *ob/ob (C57BL6)* deficient in leptin aging 8 weeks old (LIM-07) were randomly divided into two groups: sedentary (SED, n = 6) and trained (TF, n = 6). The mice were kept in standard animal facility conditions. The mice were housed in a temperature-controlled environment (22 ± 2 °C) with a 12-h light/12-h dark cycle and free access to tap water and food (Nuvilab—Nuvital Nutrientes S/A, Brazil).

### Physical training

Trained mice were submitted to AET as previously described by Ferreira et al. [27]. Before starting the training, we conducted the capacity test where we placed the mice on the treadmill, applied the speed of 0.4 km/h, and increased the speed by 0.2 km/h every 3 min until the mice got to exhaustion, which characterizes the maximal running capacity. After four weeks, we re-evaluated each mouse, and the speed for the next week was corrected. The protocol training was conducted 5 × per week, at 60% of their max capacity, without inclination, for 60 min during eight weeks. Sedentary mice were placed on the treadmill but with the treadmill off. Forty-eight hours after the last session of the training protocol, we collected the skeletal muscle. We evaluated the citrate synthase activity to certify whether the moderate training-induced metabolic modulation.

### Euthanasia

Forty-eight hours after the last training session, the mice were anesthetized with a dose of intraperitoneal ketamine hydrochloride (0.5 mL/kg), and samples were collected by exsanguination [28].

### Citrate synthase activity

Citrate synthase activity was performed as described by Le Page [29]. Briefly, the mixture containing TrisHCl (pH = 8.2), magnesium chloride (MgCl), ethylenediaminetetra-acetic acid (EDTA), 0.2–5.5 dithiobis (2-nitrobenzoic acid) (E = 13.6 mmol/(ml cm), 3 acetyl CoA, 5 oxaloacetate and 0.3 mg/ml of sample. The enzymatic activity was evaluated at 412 nm for 2 min at a temperature of 25 °C. The data was expressed as U/mg of protein.

### Lipid peroxidation assay

Lipid peroxidation was also evaluated through the substances reactive to Thiobarbituric Acid (TBARS), as described by Buege and Aust [30]. Briefly, 300 µg of protein were mixed with 30% (w/v) trichloroacetic acid and 10 mM TRIS buffer (pH 7.4) in equal volumes. After centrifuging equals volumes of samples and thiobarbituric acid was mixed, followed by boil at 100 °C for 15 min. The pink pigment formed can be evaluated at 535 nm and expressed as mM/ mg protein [30].

### Protein oxidation

Carbonyl was evaluated using the procedures described by Reznick and Packer [31]. Three hundred µg of protein was added to 30% (w/v) TCA and centrifuged at 1.180 g at 4 °C for 14 min. The pellet was resuspended in 10 mM 2, 4-dinitrophenylhydrazine and incubated in a dark room for 1 h with shaking every 15 min. The samples were washed and centrifuged three times in ethyl acetate buffer 1:1 ratio, and the pellet was resuspended in 6 M guanidine hydrochloride incubated for 30 min at 37 °C. The samples read at 370 nm and expressed as µmol/ mg protein [31].

### Superoxide dismutase activity (SOD)

SOD activity was determined according to Misra and Fridovich [32]. Samples (300 µg/protein) were incubated in 0.05 M of carbonate buffer with EDTA (pH 10.2) at 30 °C following the addition of 150 mM epinephrine. The decrease in absorbance was monitored for 1.5 min at 480 nm and the results were expressed in U/mg protein [32].

### Catalase (CAT)

CAT activity was conducted as described by Aebi [33]. Three hundred µg of protein supernatant was used in a medium containing 50 mM phosphate buffer (pH 7.0) and 0.3 M of hydrogen peroxide. The assay was monitored at 240 nm for 3 min at 20 °C, and the results were expressed as U/ mg protein [33].

### Glutathione S transferase (GST)

The activity of GST was performed as described by Habig et al. [34]. Two hundred μg of protein was added to a 0.1 M phosphate buffer (pH 6.5) containing 1 mM EDTA. Then, 60 mM of reduced glutathione and 30 mM of 1-chloro-4, 4-dinitrobenzene were added to start the reaction, which was followed at 340 nm for 1 min [34]. The results were expressed in U/mg protein.

### Reduced glutathione (GSH)

Reduced glutathione was assessed as described by Hissin and Hilf [35]. The samples were incubated in 0.1 M phosphate buffer containing 5 mM-EDTA (pH 8.0) plus 1 mg/ml of o-phthaldialdehyde (OPT) at room temperature for 15 min. Then, their fluorescences were measured at 350 nm excitation and 420 nm emission [35].

### Total sulfhydryl

The total and protein-bound sulfhydryl group contents were determined as described by Aksenov and Markesbery [36]. The reduction of 5, 5-dithiobis (2-nitrobenzoic acid) by thiol groups was measured in homogenates of 200 mg PFC, resulting in the generation of a yellow-stained compound, TNB, whose absorption is measured spectrophotometrically at 412 nm [36].

### RNA isolation and gene expression by RT-PCR

After tissue pulverization (50 mg), total RNA was prepared using Trizol® reagent (Invitrogen Life Technologies, Carlsbad, CA, USA) according to the manufacturer’s recommendations [37]. Total RNA was dissolved in RNase-free water and its integrity was checked in the 260/280 nm ratio. Samples with a ratio > 1.8 were kept at – 80 °C until processing by Reverse Transcription Quantitative Polymerase Chain Reaction (RT-PCR) analysis.

From the RNA extracted, we evaluated: PGC-1α, SIRT*-1*, ATP5A, and B2M (Table 1), through the Rotorgene 3000 (Corbett Research, Sydney, Australia) using Superscript™ III Platinum® One-Step Quantitative RT-PCR System (Invitrogen Life Technologies, Carlsbad, EUA). The cycle threshold (CT) of each targeted gene was compared with the CT of internal control and mRNA content was normalized by the 2^−ΔΔCt^ formula [37].

**Table 1:** Primer sequences

Gene name	Forward primer sequence	Reverse primer sequence
β2M	TGACCGTGATCTTTCTGGTG	ACTTGAATTTGGGGAGTTTTCTG
PGC-1α	AACAGCAAAAGCCACAAAGA	AAGTTGTTGGTTCTTGA
SIRT-1	CACAGCAAGGCGAGCATAAA	GGCAGACAATTTAATGGGGTGAA
ATP5A	TCCCTGAACTTGGAACCCGA	GGCATTTCCCAGGGCATCAA

### Statistical analysis

The data had their distribution checked through the Shapiro–Wilk test, with a normal distribution. Then, the differences between groups were compared by using the Student t-test, and the data was expressed in mean ± SEM. A *p*-value of less than 0.05 was considered statistically significant, *prism. V6* (Graph Pad Software Inc, San Diego, USA).

## Results

**Fig. 1:**
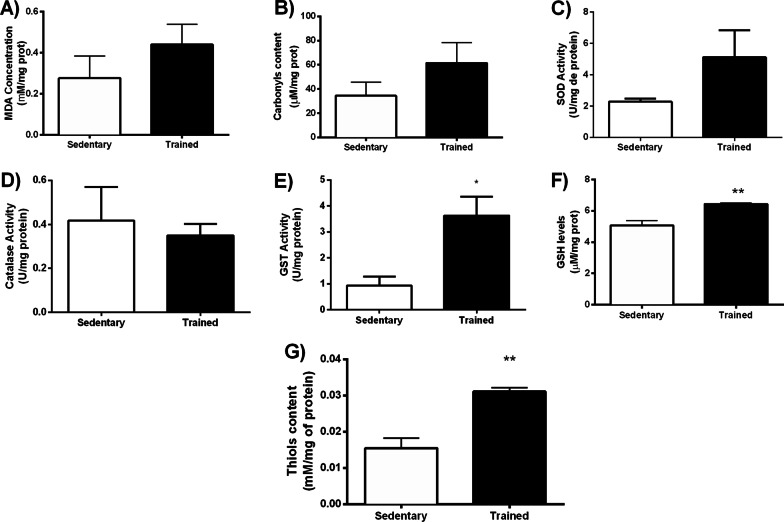
Oxidative stress biomarkers in the PFC of ob/ob mice after eight weeks of AET. **A** MDA levels, **B** Carbonyl levels, *p* = 0.2855 and *p* = 0.2246 respectively. Enzymatic antioxidant defense in the PFC of ob/ob mice after eight weeks of AET. **C** Superoxide dismutase—SOD activity, **D** Catalase—CAT activity and **E** Glutathione-S-transferase—GST activity, *p* = 0.1595, *p* = 0.6882 and *p = 0.01 respectively. Non-enzymatic antioxidant defense in the PFC of ob/ob mice after eight weeks of AET. **F** Reduced glutathione (GSH) concentration, and **G** Total Thiols levels **p = 0.005; **p = 0.0016, respectively. Non-enzymatic antioxidant defense in the PFC of ob/ob mice after eight weeks of AET. **A** Reduced glutathione (GSH) concentration, and **B** Total Thiols levels **p = 0.005; **p = 0.0016, respectively. Sedentary (*n* = 6) and Trained (*n* = 6)

Initially, we measured peak velocity by incremental exercise test in sedentary and trained obese mice, with no difference between groups (SED: 1.00 ± 0.28 vs. TF: 1.17 ± 0.4 km/h, *p* = 0.17). However, after eight weeks of aerobic training, obese trained mice showed higher peak velocity than sedentary obese mice (SED: 0.80 ± 0.2 vs. TF: 1.26 ± 0.3 km, *p* = 0.01), demonstrating a greater capacity for racing. Our training also improves the activity of citrate synthase, a marker for training adaptation in skeletal muscle (SED: 19.28 ± 0.88 vs. TF: 26.91 ± 1.12 U/mg of protein, *p* = 0.0006). Our results demonstrate that exercise does not modulate oxidative stress biomarkers in the PFC of ob/ob mice, wherein no significant differences in both MDA (SED: 0.276 ± 0.10 µmol/ mg protein vs. TF: 0.437 ± 0.09 mM/mg protein, *p* = 0.2855) (Fig. 1A) and carbonyl (SED: 34.39 ± 11.38 µmol/mg protein vs. TF: 61.52 ± 16.97 µmol/mg protein, *p* = 0.2246) (Fig. 1B) levels were found.

Although the exercise had not changed the oxidative stress-induced damage, it increased the overall antioxidant capacity. In the enzymatic system, while SOD (Fig. 1C) and CAT (Fig. 1D) activities remained unchanged, the exercise increased the GST activity (SED: 0.92 ± 0.34 U/mg protein vs. TF: 3.63 ± 0.73 U/mg protein; *p* = 0.04) (Fig. 1E). In addition, the non-enzymatic antioxidant components showed to be more responsive to exercise by increasing GSH content in 26% (SED: 5.07 ± 0.29 µmol /mg protein vs. TF: 6.43 ± 0.06 µmol/mg protein; *p* = 0.001) and the sulfhydryl groups in 66.7% (SED: 0.01 ± 0.002 mmol/mg protein vs. TF: 0.03 ± 0.001 mmol/mg protein; *p* = 0.02), Fig. 1F and G, respectively.

**Fig. 2:**
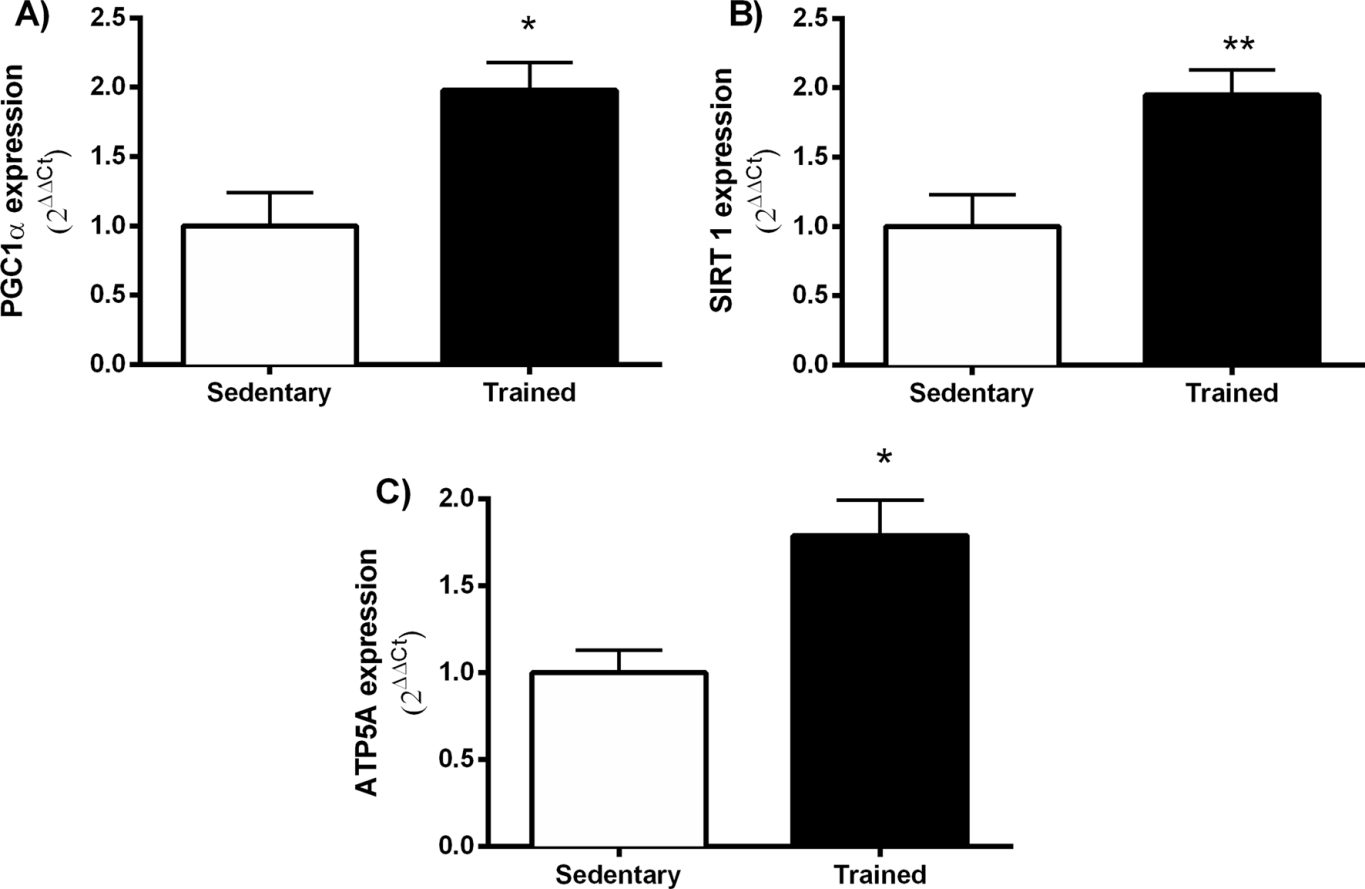
Evaluation gene expression of ob/ob mice after eight weeks of AET in the PFC. **A** Peroxisome proliferator-activated receptor gamma coactivator 1-alpha (PGC-1α), **B** Sirtuin-1 (SIRT-1) and **C** Adenosine tri phosphate synthase 5A gene (ATP5A). *p = 0.01; **p = 0.009 and *p = 0.02, respectively. Sedentary (*n* = 6) and T: trained (*n* = 6)

Additionally, evaluating genes involved in mitochondrial biogenesis, we demonstrate that AET up-regulates the expression of Peroxisome Proliferator-Activated Receptor Gamma Coactivator 1-alpha (PGC-1α) (*p* = 0.01) and Sirtuin-1 (SIRT-1) (*p* = 0.009), Fig. 2A and B, respectively. Furthermore, gene expression of the Adenosine Tri Phosphate Synthase 5A (ATP5A), which encodes the ATP synthase into the mitochondrial electron transport chain, was also increased following AET (*p* = 0.01) (Fig. 2C).

## Discussion

The PFC, located in the front region of the brain, is implicated in several internal desires. It is the area responsible for executive function associated with the decision-making processes related to the choice between good and bad, conflicting thoughts, judging future consequences of present actions, and working regarding ambitions, prediction, and expectancies[38, 39]. In individuals with the normal function of the PFC, the ability to exert full control over the dietary desires for fatty and sugary food are effective, demonstrating the capacity for self-regulation/self-control expectancies [38, 39].

Some models of obesity demonstrate reduced signaling in PFC, and corroborative studies have also shown that increased activity in a specific region of the PFC moderates food cravings and consumption of these hyper-palatable foods, whereas the reduced activity of the same region can increase food consumption [40–42].

All regions in the brain are highly susceptible to reactive species (RS), especially due to the ATP-required high consumption of O_2_, amounts of excitatory amino acid transmitters and, calcium metabolism. Thus, the inappropriate balance between the production and removal of RS, and the mitochondrial dysfunction-related energy supply, might deregulate the whole brain function. This is an important concept because mitochondria play a fundamental role in the life and function of several cells in the brain, and cellular injury that impairs the capacity to generate ATP leads to cell death [43]. Mitochondria are dynamic organelles the function are modulated by the energy needs of tissues [44]. Exists a fine adjustment between nuclear and mitochondrial gene expression that controls the assembly of the mitochondrial respiratory complex; therefore, the energetic demand controls phosphorylative capacity and the ATP supply [44]. In conditions of exercise, wherein exist a high energetic demand, mitochondrial biogenesis is triggered, activating the signaling cascade related to the SIRT-1, PGC-1α and nuclear respiratory factor-1/2 (NRF1/2), increasing the accurate communication between the nucleus and mitochondria to produce more mRNAs and mitochondrial proteins [45]. In turn, these increases in mitochondrial content, number, and energy demands need also to regulate the REDOX balance in mitochondria to maximize the capacity of mitochondria to perform oxidative phosphorylation without an increase in RS leaking or a decrease in antioxidant defense [46].

In some neurodegenerative diseases, mitochondrial damage reduced oxidative phosphorylation capacity, increases RS production, leading to oxidative stress and negatively influencing brain function [47–49]. Data in the literature demonstrated the involvement of RS in alternating the control of satiety and hunger behavior [50, 51]. Specifically, in PFC, previous data in the literature demonstrate that obesity induced by high-fat diet, results in an increase of RS, oxidative stress biomarkers, and mitochondrial dysfunction [52, 53].

In our study, we demonstrated how exercise affects oxidative balance and mitochondrial biogenesis in the PFC of obese mice. Our findings demonstrate that AET improves antioxidant defense while up-regulates mitochondrial biogenesis and ATP synthase expressions. Our data showed that AET for 8 weeks improves GST activity, GSH, and total thiol levels, leading to an increase in antioxidant defense after 8 weeks. The activity of the glutathione pathway is mediated by tissue levels of reduced glutathione associated with the action of the glutathione reductase. This enzyme is associated with the plasma membrane participating in converting of GSSG to GSH through the oxidation of electron carriers, including nicotinamide adenine dinucleotide phosphate in its oxidized and reduced form (NADP^+^ and NADPH). These reactions are essential for the decrease of oxidative damage that affects cellular components participating in the removal of ROS and metabolic detoxification [54, 55].

Neves et al. [56] demonstrated that AET applied for 8 weeks decreased OS associated with increased antioxidant defenses in these CNS tissues. In addition, Aksu et al. [57], similarly, evaluated the acute and chronic effects of AET on the PFC, hippocampus, and striatum, their results showed that AET does not induce OS in these different brain areas [58]. These results corroborate with Flôres et al. [59] where the group showed that 12 weeks of AET increases GSH levels in PFC. Recently Comim et al. [60] showed that low-intensity training for 8 weeks was able to reverse the impairment in memory and learning, in addition to the decrease in oxidative stress biomarker in encephalic tissues, including PFC.

In addition, seeking to elucidate the possible interaction between antioxidant defense and OS biomarkers with other components of oxidative metabolism, we also evaluated the expression of important mitochondrial transcription factors, PGC-1α and SIRT-1 [61]. In this sense, it was that the trained group had increased both genes compared to the sedentary group. Contributing to our findings, Steiner [62] and colleagues performed 8 weeks of treadmill running (1 h/day, 6 days/week at 25 m/min and 5% incline), and showed increases in gene expression of PGC-1α and SIRT-1 in the brainstem, hippocampus and hypothalamus. Additionally, recent studies have shown that the contraction of skeletal muscle in response to aerobic exercise can activate the signaling pathway SIRT-1 / PGC-1α / Fibronectin type III domain-containing protein 5 (FNDC5) in the central nervous system, the precursor of irisin through metabolites including interleukin-6 and lactate that cross the blood–brain barrier promoting neurotrophic responses in different brains [63–65]. Supporting our data related to PGC-1α and SIRT-1 and the fact of AET may induce mitochondrial biogenesis, we quantify the levels of ATP5A, an important subunit of ATP synthase that can be used as an indicator of mitochondrial biogenesis. Our data showed that the levels of ATP5A in the trained group were higher than the control, showing that exercise is capable to induce mitochondrial subunits biogenesis. Contributing to our data, Braga et al. [66] showed that exercise during 5 week, one hour per day, 5 days per week at 60% of maximal capacity promoted an increase in the gene expression of OXPHOS subunits encoded by nDNA (Atp5a) in the lateral hypothalamus.

Taken together our data with previous data in the literature we can speculate that physical exercise can activate a complex communication between central and peripheric tissues leading an increase in brain metabolism [64, 67]. In a future study, we plan to evaluate the OXPHOS subunit’s function, protein levels, and mitochondrial respiration capacity to direct link aerobic training with an improvement in PFC function from obese individuals. Raising the relevance of physical training as a therapeutic strategy to combat the global epidemic of obesity.

## Conclusion

In summary, our study demonstrated that AET minimizes the effects of the obesity-induced OS in the PFC by activating antioxidant defenses and mitochondrial transcript factors, that can improve mitochondrial biogenesis and function.

## Data Availability

The datasets used and/or analysed during the current study are available from the corresponding author on reasonable request.

## Abbreviations

AET: Aerobic physical training
ATP: Adenosine tri phosphate
ATP5A: Adenosine tri phosphate synthase 5A
CAT: Catalase
CNS: Central nervous system
FNDC5: Fibronectin type III domain-containing protein 5
GSH: Reduced glutathione production
GST: Glutathione S Transferase
HII: High-intensity interval aerobic physical training
MDA: Malonaldehyde
OS: Oxidative stress
O_2_: Oxygen
PFC: Prefrontal cortex
PGC-1α: Peroxisome proliferator-activated receptor gamma coactivator 1-alpha
RT-PCR: Quantitative polymerase chain reaction
ROS: Reactive oxygen species
SED: Sedentary group
SOD: Superoxide dismutase
SIRT-1: Sirtuin-1
TBARS: Thiobarbituric acid
TF: Trained group
